# Parents’ perceptions of the impact of the novel coronavirus (COVID-19) on the eating behaviors and routines of children with autism spectrum disorders (ASD)

**DOI:** 10.3389/fpsyt.2024.1296643

**Published:** 2024-03-28

**Authors:** Mudi H. Alharbi

**Affiliations:** Clinical Nutrition Department, College of Applied Medical Sciences, Taibah University, Madinah, Saudi Arabia

**Keywords:** autism spectrum disorder (ASD), coronavirus, COVID-19, eating behaviors, routines, parents

## Abstract

**Background:**

Restricted interests and repetitive behavior are characteristics of autism spectrum disorder (ASD). The likelihood that persons with ASD will respond adversely to unfamiliar situations is great. The novel coronavirus outbreak has resulted in disruptions to all aspects of routine and behavior. Hence, this study proposed to investigate the impact of the outbreak on the eating behavior and routines of children with ASD in Saudi Arabia through the perceptions of their parents.

**Method:**

A cross-sectional study with a quantitative approach was utilized to obtain data from 150 parents of children with ASD aged ≤18 years in Saudi Arabia. The data collected included demographic data of the parents, the ASD status of the family, impact of COVID-19 to the family, eating behavior of the children with ASD, and daily routines of the children with ASD. Moreover, parents were able to provide comments regarding their children’s eating behavior or daily routines.

**Results:**

The study found that changes in the eating behavior of children with ASD were found to differ significantly (p<0.05) based on the number of children with ASD, the age of the children with ASD, the gender of the children with ASD, and the severity of their ASD symptoms. Moreover, changes to dinner-time routines were found to differ significantly (p<0.05) based on the age of the children with ASD. Also, changes to morning routines were found to differ significantly (p<0.05) based on the age of the children with ASD, their gender, and the severity of their ASD symptoms. Additionally, impact of COVID-19 to the family had a significant impact to eating behavior and daily routines of the children with ASD.

**Conclusion:**

This study found that the eating behavior and daily routines of children with ASD in Saudi Arabia have been considerably worsened and changed. The study recommends the collaboration of multidisciplinary teams and parents to modify or design interventions that help to change their eating behavior and routine can be implemented in the home. It also recommends the provision of virtual helplines to aid parents of children with ASD in such cases.

## Introduction

As with other countries across the globe, the Kingdom of Saudi Arabia (KSA) is in the grip of the COVID-19 pandemic. Even before its first case was confirmed on March 2, 2020, the country took several measures to restrict the spread of the disease within the kingdom (1). Eventually, schools and universities were closed on March 8, 2020, together with a ban on gatherings (2). All educational institutions were included in this decision such as, private and public schools, and establishments for vocational and technical training. In their place, online teaching and home-schooling were encouraged (1). Prior research has indicated that reactive closure of schools can dilute the system of societal dealings, diminish and defer the peak of an endemic, and lessen the propagation of influenza, whether regular or epidemic (3, 4).

These measures caused a sudden change of routine for children and their parents as they are required to stay at home in response to the government’s directives for social distancing and the closure of schools. In this context, dealing with children with special needs becomes challenging for their families and caregivers. In particular, children with autism spectrum disorder (ASD) experience a considerable change in their routines. For instance, such children typically have several intervention sessions in school, with specialist therapists, or in clinics and institutes dedicated to this purpose. However, due to the measures to contain the contagion, children with ASD have encountered significant disruption to their schedules (5).

Persons with ASD often require long-term support (6) since their symptoms impact day-to-day existence (7). Moreover, their capacity to meet the demands of new situations, learning, and problem-solving are greatly limited (8). Further, there is a greater likelihood that persons with ASD will experience anxiety and depression (9, 10), which may be exacerbated by unfamiliar situations, such as a quarantine (7).

An associated concern for children with ASD is their eating behavior, as many persons with ASD are hypo- or hypersensitive to taste and touch. This indicates that they may be either oversensitive or impervious to the temperature or texture of foods and consequently finicky about food. Consequently, their diet may be restricted to certain foods or specific brands (11). Thus, restricted preferences for food (also termed selective eating, restricted variety, food selectivity, or limited food repertoire) are the problem most frequently associated with feeding in children with ASD (12–14). Prior research in an Omani context has also reported the high occurrence of food selectivity and refusal in children with ASD (15). Moreover, the findings of Vissoker et al. (16) confirmed earlier research on the greater prevalence of eating rigid patterns and eating problems in children with ASD. Further, they highlighted the role of age in this regard. For instance, food selectivity, rituals, and sameness increased with increase in age (16). On the other hand, Patton et al. (17) found that lower consumption of unfamiliar foods, a higher number of instances of behavior that disrupted mealtimes, and a higher number of commands from parents to remind children to take bites during meals were associated with greater severity of ASD (17).

Moreover, in their exploration of the mealtime behavior of children with ASD in the school setting, Padmanabhan and Shroff (18) found that such children struggled at mealtimes if the food was not to their liking, if the food was not what they preferred to eat routinely, and if there had been any change to the schedule of the break times. Further, sensitivity to the smell of food and loudness of others during break times prevented them from consuming their meals. Another facet was related to their tactile sensitivity. That is, they would not eat if the texture of the food was not to their liking (18). Huxham et al. (19) also drew attention to the preference of children with ASD for foods of a certain appearance, for instance according to food color, food presentation and the brand and packaging of food (19). Acceptance of food was also affected by the children’s sensory features and food texture. Moreover, Mayes and Zickgraf (20) reported that children with ASD had a greater likelihood of atypical eating behavior than children with ADHD or other disorders, or typically developing children (20). Atypical eating behavior included restricted preferences for food, hypersensitivity to textures of food, other odd patterns such as eating only a certain brand of food, pocketing the food instead of swallowing it, and pica. In the present spread of several epidemics, it is possible that certain foods (or brands of food) that they eat daily may not be easily accessible, which can lead to disturbances in their eating behavior (11).

In addition, Altable (11) emphasized the criticality of routines and control to persons with ASD as they can be disturbed by the minutest and most commonplace alterations (11). Thus, in the context of the COVID-19 pandemic, where routines have been impacted, there is a great likelihood that the daily routines of children with ASD will have been impacted in different ways (11). Eshraghi et al. (21) also highlighted the preoccupation with routine of children with ASD and the high probability of upheaval (emotional and behavioral) due to COVID-19 (21). In an Australian study, Marquenie et al. (22) found that the dinner-time routines of families with young children (2–5 years) with ASD were chaotic and unstructured (22). In contrast, bedtime routines were more structured and, often, non-functional. Moreover, an investigation by Colizzi et al. (23) revealed that various new needs emerged in persons with ASD due to the COVID-19 outbreak (23). For instance, they required greater healthcare support and in-home support, in particular, together with interventions to deal with the disruption caused by the quarantine. Moreover, difficulties in coping with daily activities increased and behavior problems presented more frequently or intensively in one of out of three children with ASD as a minimum. A study conducted in Italy by degli Espinosa et al. (24) highlighted how behavioral support and reinforcement for children with ASD could be provided at home by their parents during the pandemic (24). On the other hand, Stankovic et al. (25) explored the challenges encountered by the parents of children with ASD in Serbia during the COVID-19 situation and found that the absence of support and feelings of helplessness had intensified during this time (25).

### Food selection and preferences in ASD

A common related facet of children with ASD, which affects 46% to 89% of such children, is eating challenges (26, 27). Sharp et al. (28, 29) reported that the probability of children with ASD experiencing a feeding problem was five times greater than of children without ASD. Research has indicated that these feeding problems could be a demonstration of the limited interests and activities characteristic of children with ASD (26). A further explanation could be that the behavior of the family could influence the feeding problems of such children either via lowered exposure to a variety of foods (30) or via unintentional support of problem behaviors concerning mealtime (31).

Field et al. (32) highlighted the specific feeding problems encountered in children with ASD. These included refusal of food, selectivity of food by type, selectivity by food by texture, oral motor delays (e.g., chewing, repositioning the tongue, lip closure, etc.), or dysphagia (challenges with swallowing). Children with ASD typically exhibited selectivity by type or texture followed by oral-motor delay leading to mechanical challenges in eating foods; and dysphagia (32). Other studies have reported severe problems with behavior at mealtime (31, 33).

A variety of challenges related to mealtime and eating is experienced by these children and the resulting difficulties can result in insufficient nutrition, disruptive behaviors at mealtime, rigid food-related routines, and intensive effort from members of the family (34–36). The occurrence of disturbances to mealtimes is due to the need of the child with ASD for greater help and supervision, a distinct meal, or since the atmosphere at mealtime is stressful as a result of the extent of attention required by the child with ASD (37).

Ausderau and Juarez (38) noted the commonness of feeding disorders in children with ASD resulting in considerable impacts to their family mealtimes. The study found that while families gave importance to mealtimes, these were often not easy to structure and frequently resulted in the mother’s exhaustion. Moreover, the children with ASD exhibited unusual preferences for food, food selectivity, and disruptive behaviors at mealtime (38). In another study, Aponte and Romanczyk (39) examined the association between feeding problems and autism severity. They found that various feeding problems and the duration of negative vocalizations during observations of meals were predicted by autism severity.

BalikçCheck that all equations and special characters are displayed correctly.i and Çiyiltepe (40) used the BAMBI (Brief Autism Mealtime Behavior Inventory) (41, 42) to study the feeding problems of children with ASD. Their study found that the feeding problems exhibited by children with ASD included behavioral problems at mealtime, such as sobbing and screaming throughout meals followed by avoidance of certain food types and textures, selectivity of type and texture, and dislike of some food types and textures (40). In another study, Bandini et al. (43) found that in contrast to children without ASD, children with ASDs displayed more food refusal and more limited repertoire of food. A later study by Bandini et al. (14) evaluated food selectivity of children with ASD in a longitudinal study. Overall, an improvement in food refusal could be seen between baseline and follow-up. However, the food repertoire namely, number of distinctive foods partaken, did not seem to increase.

In a Turkish study, Bicer and Alsaffar (12) studied the dietary intake and feeding problems of 164 children (aged 4–18 years) and reported that the most typical feeding problems of these children were consuming a restricted range of foods (food selectivity), rapid eating, and overeating. Correspondingly, common strategies adopted by parents/caregivers to address these feeding problems included distraction, permitting more drinking of fluids, and offering preferred foods. Other approaches utilized included compelling, offering rewards, wheedling, child-led feeding, giving meals a miss, chastisement, and utilizing high-calorie supplements/formula.

Mahmoud et al. (44) contrasted the feeding behavior of 35 children aged 2 - 4 years recently diagnosed with ASD with 70 children who were typically developing (TD). This study reported that children with ASD demonstrated a greater extent of challenging feeding behaviors, such as feed neophobia, consuming non-food items, needing help when eating, and avoidance of food of certain taste or texture (44). Furthermore, Gray and Chiang (45) reported that the problematic mealtime behaviors exhibited by Chinese-American children with ASD included preference for certain food textures (e.g., crunchy), unwillingness to try new foods, and inability to stay seated at the table till the end of the meal.

### Determinants of feeding difficulties

Various facets have been recognized to influence feeding difficulties of children with ASD. These include age, ASD severity, and ASD symptoms, (46) among others. The relationship between age of a child with ASD and difficulties in feeding has been studied by various researchers with two reporting that these were negatively related (14, 47), that is, eating difficulties may diminish as the child grows older; one reporting a favorable relationship (16); and three reporting no relationship (48–50). On the other hand, concerning the relationship between ASD symptoms and their severity, with difficulties in feeding, researchers have reported favorable or no relationship. For example, Pham et al. (51) reported a positive association whereas Prosperi et al. (52); Sharp et al. (28, 29); and Smith et al. (50) reported that there was no relationship between feeding difficulties and ASD symptoms and their severity. Moreover, while Pham et al. (51) noted that the incidence of food selectivity corresponded to increased ASD severity, that is, to severe from moderate and to moderate from mild. In contrast, some other studies (13, 53) reported that the method of assessment, such as the usage of Autism Diagnostic Interview-Revised (ADI-R) (54), Autism Diagnostic Observation Scale-Calibrated Severity Scale (ADOS-CSS) (55), Social Responsiveness Scale (SRS) (56), BAMBI, among others, influenced the relationship between feeding difficulties and severity of ASD symptoms.

The role of gender has also been studied, though not specifically. For instance, Seiverling et al. (57) reported that boys with ASD were more likely to have feeding challenges than girl children with ASD. Leader et al. (58) also reported that gender was significantly associated with food selectivity. On the other hand, Worley and Matson (59) found that there the differences among the genders was not significant for eating problems, such as over/under eating. Also, Babinska et al. (60) found that food selectivity, problems at mealtime, diet, and usage of food supplements had no interactions with gender. That is, high incidences of challenging behavior associated with food intake were seen in both genders regardless of age. However, severity of symptoms was found to be correlated to challenging eating behavior.

### Routines and ASD

Routines are defined as “observable, repetitive behaviors which directly involve the child and at least one adult acting in an interactive or supervisory role, and which occur with predictable regularity in the daily or weekly life of the child” and “may occur at a regular time, in the presence of a regular adult, in a regular place, in a regular sequence, or a combination of these” (61, p. 243). Routines are considered to be essential in establishing the basis for development of ritual. Rituals, in their turn, are regarded as significant in developing a family unit that is robust and healthy (22). In families of children with ASD, the daily routines are often centered around the characteristic demands of the child rather than those of the family in its entirety (62). Moreover, children with ASD can frequently find it difficult to participate in daily routines in their home, such as bath or bed time (63). Boyd et al. (64) reported that there were some common facets of research related to family routines and families of children with ASD. The facets were stress related to the necessity for and challenges with routines, the connection between family routines and the family’s health, participation of the family as planned around the child with ASD, adherence to routines while encountering challenges, cumbersome routines, significant routines for bonding of the family, and adaptations made by families to routines. Henderson et al. (65) found that quality and hygiene of sleep were associated with not only routines but also externalizing behaviors in a group of 58 children with ASD.

Marquenie et al. (22) found that families of children aged 2-5 years with ASD experienced dinnertime routines that were unruly and unstructured. In contrast, bedtime routines involved routines that were more structured and often non-functional. Thus, in contrast to dinnertime routines, bedtime routines were involved significant rituals and interactions. In another study, Ibañez et al. (63) used a randomized controlled trial to scrutinize the effectiveness of an interactive, web-based tutorial for parents in enhancing the engagement of children with ASD (aged 18-60 months) in everyday routines. This study found that the use of such a tutorial enhanced child participation in routines.

Stoppelbein et al. (66) used the Child Routines Questionnaire (CRQ) (61) to compare details of the routines of 45 children with ASD and 45 typically developing children of matching ages/genders. The parents of the children in the ASD group were found to report fewer routines. Moreover, children with ASD were found to have greater internalizing and externalizing symptoms than the children without ASD. In addition, the routine levels of younger children with ASD were found to be lower than those of older children (66). Mirzaie et al. (67) reported that it was difficult for families of children with ASD to follow routines due to different reasons, such as the children’s lack of flexibility, challenging behaviors, problems with sensory-processing, anxiety or marital issues of parents, and environmental aspects including poor access to ASD-related healthcare services in areas that were remote and less populated. McAuliffe et al. (68) highlighted that the efforts needed to develop family routines were substantial and that these could be at the detriment of the health and wellbeing of mothers of children with ASD.

### Impacts of COVID-19

COVID-19 disrupted routines, school activities and special programs, day programs, and also support at work (11). Baweja et al. (69) reported that the different challenges encountered by persons with ASD during the COVID-19 pandemic included disruptions owing to changes in education and vocation, challenges to routines associated with home and leisure, restricted obtainability of behavioral health services, and modifications in delivery of health services. Individuals with ASD were more vulnerable due to their characteristics and typically accompanying conditions (69).

Narzisi (5) highlighted that children with autism spectrum conditions (ASC) typically have interventions for many hours a week, either at their homes with trained therapists or in assigned institutions or hospitals. Nevertheless, due to the COVID-19 pandemic, the physical support provided by therapists to these children and their families could not be continued. Moreover, they could not go to external interventions (5). Persons with ASD typically receive therapy in different ways (e.g., speech, physical, behavioral, social, occupational, and psychological). However, with COVID-19, persisting with these therapies was largely impossible (11). Manning et al. (70) found that the predominant facets of stress in families of persons with ASD were related to disruption of therapeutic service, finances, and illness. Moreover, higher stress was reported for caregivers of persons who had received a high intensity of services prior to COVID-19. The main concerns voiced by the respondents were related to obtaining respite care during the pandemic (70).

### The present study

The aim of this study was to investigate the eating behavior and routines of children with ASD in Saudi Arabia during the COVID-19 outbreak. Two research questions informed the study: (1) What is the impact of COVID-19 on the eating behavior of children with ASD in the KSA? and (2) What is the impact of COVID-19 on their daily routines? Based on these research questions, the study hypothesized that the eating behavior of children with ASD and their daily routines could have significantly worsened in comparison to their typical state prior to the closure of schools. This study is significant because it endeavors to draw attention to the current status of children with ASD in the KSA in this regard and to provide insights for appropriate supportive action.

**Figure 1:**
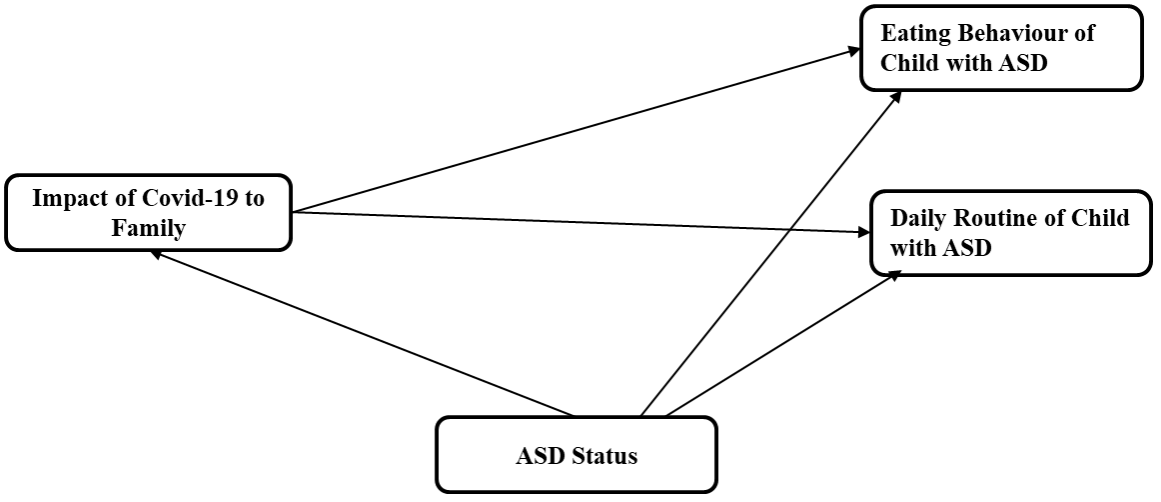
Conceptual model.

The scrutiny of prior research suggested that the pandemic would impact eating behavior and routines of children with ASD. The impact of different determinants (e.g., Age, Gender, and Severity of ASD symptoms) on children’s difficulties with feeding was another facet that has been explored in prior literature (46) and was thus included in the study for scrutiny extending the consideration to routines as well. **Figure 1** depicts the proposed conceptual model for the study.

## Method

### Measures

A total of 150 Saudi parents of children with ASD were invited to participate in this survey as part of a cross-sectional study. The children described in the study were aged ≤18 years. The survey or questionnaire was distributed using email by contacting different Autism institutions. A questionnaire was designed in English and subsequently translated into Arabic. The following data were collected in the period April, 2020 to May, 2021:

(1) demographic data of the parents such as gender, age, marital status, educational qualifications, family income, current working situation (6 items);
(2) family ASD status such as number of children with/without ASD, age of children with ASD, gender of children with ASD, severity of ASD symptoms (5 items);
(3) impact of COVID-19 restrictions on family (10 items) adapted from COVID-related studies (23, 70). This scale used a five-item Likert scale: 1-No impact; 2-Little impact; 3-Impacted to some extent; 4-High impact; 5-Very high impact;
(4) eating behavior of children with ASD (12 items) adapted from the findings of various studies e.g., (20). This scale used a five-item Likert scale: 1–Considerably improved, 2–Slightly improved, 3–No different, 4–A little worse, and 5–Very much worse; and
(5) daily routines of children with ASD (16 items) modified from the Daily Routine and Autonomy (DRA) questionnaire and the findings of other studies e.g., (22, 71). The DRA questionnaire was developed by Lamash and Josman (71) in acknowledgement of the limited independence displayed by persons with ASD in daily activities. This study utilized sections of the questionnaire that were deemed suitable for a wider sample of children with ASD. Again, this scale used a five-item Likert scale: 1–Considerably improved, 2–Slightly improved, 3–No different, 4–A little worse, and 5–Very much worse.

At the end of the questionnaire, space was provided for the parents to add further comments or notes related to their children’s eating behavior or daily routines. This study was carried out in accordance with the Declaration of Helsinki and with the approval of ethics committee of applied medical sciences at Taibah university no, 2020/52/201/CLN.

### Statistical analyses

Children’s ASD behaviors during the COVID-19 outbreak were rated by their parents using the Arabic or English version of the questionnaire. The validation was assessed. The study utilized different statistical analyses such as frequencies and percentages, descriptive statistics (mean (M) and standard deviation (SD)), and inferential statistics (one-sample t-test, one-way ANOVA, and regression). These analyses served to assess the changes to eating behavior and daily routines of the children with ASD. Moreover, the impact of the family ASD status on eating behavior and daily routines was also analyzed. All tests were performed using SPSS software version 26.0. The questionnaire was presented to a group of specialists in the field of nutrition and special education in ASD. Hence, the internal consistency coefficients were extracted for the overall instrument and for each individual domain using Cronbach’s alpha formula. The value of the alpha coefficient for the instrument as a whole unit was 0.85. The value of the alpha coefficient was 0.84 for the first domain (eating behavior), and 0.86 for the second domain (eating routines).

## Results

### Demographic information

The majority of the participating parents were female (66.0%) and aged between 21 and 30 years (50.0%). Moreover, the majority of the parents were still married (81.3%), were graduates (69.3%), and had a family income between 10,000 and 15,000 SAR (46.7%). Further, 8% were not working due to COVID-19, having lost their jobs during the pandemic, indicating that the pandemic had impacted the livelihood of these parents. A large proportion of the participating parents (70.7%) were able to work from home. The demographic details of the parents are summarized in **Table 1**.

**Table 1:** Parents’ Demographic information.

Demographics	Categories	Frequency	Percent
Gender of parent	Male	51	34.0
	Female	99	66.0
Age (Years)	21-30	75	50.0
	31-40	61	40.7
	41-50	14	9.3
Marital Status	Married	122	81.3
	Divorced	28	18.7
Educational Qualification	High School or diploma	41	27.3
	Graduate	104	69.3
	Post-graduate	5	3.3
Family income	<5000 SAR	2	1.3
	5000 – <10000 SAR	65	43.3
	10000 – 15000 SAR	70	46.7
	>15000 SAR	13	8.7
Current working situation for the parents	Not working before Covid-19	2	1.3
	Not working due to Covid-19	12	8.0
	Working from home	106	70.7
	Commuting to work	30	20.0
**Total**		**150**	**100.0**

### Family ASD status

The majority of the participating families had one child with ASD (96.0%) and one or two children without ASD (70.0%). The ages of the children with ASD were mostly 3–5 years (52.7%) followed by ages of 6–10 years (25.3%). The majority of the children with ASD were male (62.7%). The severity of their symptoms was mostly moderate (64.0%), followed by mild symptoms (28.0%). There were also a few (8.0%) children with ASD who had severe symptoms. Also (**Table 2**).

**Table 2:** Family ASD status.

Variable	Category	Median*	Frequency	Percentage
Number of children with ASD	1	1	144	96.0
	2		6	4.0
Number of children without ASD	1	2	69	46.0
	2		70	46.7
	3		11	7.3
Age of children with ASD (Years)	≤ 2	2	18	12.0
	3 – 5		79	52.7
	6 – 10		38	25.3
	11 – 18		15	10.0
Gender of children with ASD	Male	1	94	62.7
	Female		56	37.3
Severity of ASD symptoms	Mild		42	28.0
	Moderate	2	96	64.0
	Severe		12	8.0
**Total**			**150**	**100.0**

*Age of children with ASD;2 = 3-5 years old. Gender of children with ASD: 1= male. Severity of ASD symptoms; 2= Moderate.

### Children’s eating behavior in the pandemic

A one-sample t-test, utilized to assess the parents’ perceptions of the changes to their children’s eating behavior and daily routines in the pandemic, revealed that the children’s eating behavior (M=3.805, SD=0.444) had significantly worsened in comparison to their normal state (95% CI=3.73–3.88), which was significantly higher than the rating of “3” – (Not different) with median 4 (A little worse), p<.001 (**Table 3**).

**Table 3:** Parents’ perceptions regarding changes in eating behavior of children with ASD.

	Median	Mean	SD	Sig.	95% Confidence Interval (CI) of the Difference
Eating Behavior of Child with ASD	4*	3.805	0.444	<0.001	3.7334 – 3.8766

*4=A little worse.

Further, from **Table 4** it could be seen that the three facets of eating behavior that had changed the most were “*Rejection of food based on color or shape*” (M=4.693, SD=0.655); “*Rejection of food based on texture*” (M=4.500, SD=0.833); and “*Eating only certain kinds of food (e.g., grain products, chicken, fruit, French fries, etc.)*” (M=4.313, SD=0.604). The three facets of eating behavior that had changed the least were “*Exhibiting pica (that is, eating non-food substances such as crayons, dirt, soap, etc.)*” (M=2.727, SD=1.088); “*Smelling foods before eating*” (M=2.933, SD=1.168); and “*Not mixing foods before eating*” (M=3.187, SD=0.878).

**Table 4:** Changes to eating behavior.

Statements	Median*	M	SD
Acceptance or preference of different foods	4	4.207	0.862
Rejection of food based on texture	5	4.500	0.833
Rejection of food based on temperature	4	3.733	0.711
Rejection of food based on color or shape	5	4.693	0.655
Pocketing of food without swallowing it	4	3.980	0.573
Eating only certain kinds of food (e.g., grain products, chicken, fruit, French fries, etc.)	4	4.313	0.604
Eating only certain brands of a food	4	4.047	0.698
Not mixing foods before eating	3	3.187	0.878
Smelling foods before eating	3	2.933	1.168
Using only a certain plate or cup	3	3.420	0.853
Preferring food to be presented in a specific manner	4	3.927	0.419
Exhibiting Pica (that is, eating non-food substances such as, crayons, dirt, soap, etc.)	2	2.727	1.088

*1=Considerably improved, 2=Slightly Improved, 3=Not different, 4=A little worse, 5=Very much worse.

### Children’s daily routines in the pandemic

Similarly, it could be seen that the daily dinner-time routines of the children (M=3.593, SD=0.483) had significantly worsened in comparison to their state prior to the pandemic (95% CI=3.8746–4.0304, P<0.001). Likewise, the morning (M=4.265, SD=0.513, 95% CI=4.1822–4.3478, P<0.001) and bedtime (M=3.555, SD=0.483, 95% CI=3.4771–3.6329, P<0.001) routines had also significantly worsened in comparison with their state prior to the pandemic (**Table 5**).

**Table 5:** Parents’ perceptions regarding changes in daily routines of children with ASD.

	Median*	M	SD	Sig.	95% Confidence Interval (CI) of the Difference
Dinner time routines	4	3.953	0.483	<0.001	3.8746 – 4.0304
Morning routines	4	4.265	0.513	<0.001	4.1822 – 4.3478
Bedtime routines	4	3.555	0.483	<0.001	3.4771 – 3.6329

*4=A little worse.

From **Table 6**, it could be seen that the dinner-time routine that had deteriorated the most was “*Range of foods to eat*” (M=4.313, SD=0.787), whereas the morning routine that had deteriorated the most was “*Willingness to dress*” (M=4.487, SD=0.833). The bedtime routine that had deteriorated the most was “*Willingness to keep clothes ready for the next day*” (M=4.020, SD=0.893). Overall, all the facets of the morning routine appeared to have deteriorated the most. Some facets of the dinner-time routines and bedtime routines had deteriorated slightly in comparison, such as “*Usage of preferred plates, spoons, glasses, etc*.” (M=3.547, SD=0.931) and “*Need for certain pillow/blanket*” (M=3.680, SD=0.805). Also, although the mean of the parents’ perceptions regarding the children’s “*Willingness for the light to be switched off*” indicated slight improvement to no change (M=2.593, SD=0.991).

**Table 6:** Changes to daily routine.

Statements	Median	Mean	Std. Deviation
Dinner time routines			
Range of foods to eat	4	4.313	0.787
Usage of preferred plates, spoons, glasses, etc.	3	3.547	0.931
Usage of same chair at dinner table	4	3.680	0.805
Need for same position at dinner table	4	4.160	0.696
Refusal to feed himself/herself	4	4.087	0.919
Retching when certain foods are seen/presented	4	3.807	0.808
Throwing tantrums at the dinner table	4	4.067	0.960
Eating with the family	4	3.967	0.915
Morning routines			
Willingness to brush teeth	4	4.207	0.688
Willingness to bathe	4	4.120	0.723
Willingness to go to the toilet	4	4.227	0.820
Willingness to dress	5	4.487	0.833
Bedtime routines			
Willingness to change into night clothes	4	3.927	0.419
Willingness for the light to be switched off	2	2.593	0.991
Need for certain pillow/blanket	4	3.680	0.805
Willingness to keep clothes ready for the next day	4	4.020	0.893

*1=Considerably improved, 2=Slightly Improved, 3=Not different, 4=A little worse, 5=Very much worse.

### Influence of family status on eating behavior and daily routines

As can be seen in **Table 7**, the change in the eating behavior of a child with ASD was found to differ significantly (p<0.05) based on the number of children with ASD, age of children with ASD, gender of children with ASD, and severity of their ASD symptoms. However, *change in eating behavior* did not differ significantly based on number of children without ASD. *Changes to dinner-time routines* were found to differ significantly (p<0.05) based on the age of children with ASD, but not on the number of children with or without ASD, gender of children with ASD, and severity of their ASD symptoms. *Changes to morning routines* were found to differ significantly (p<0.05) based on age of children with ASD, their gender, and the severity of their ASD symptoms, but not number of children with or without ASD. *Changes to bedtime routines* were found to differ significantly (p<0.05) based on the age of children with ASD but not with the number of children with or without ASD, gender of children with ASD, or severity of their ASD symptoms.

**Table 7:** Influence of family status on eating behavior and daily routines.

Family Status	Categories	n (%)		Eating Behavior of Child with ASD		Dinner time routines	Morning routines		Bedtime routines		
				Mean ± SD	p-value	Mean ± SD	p-value	Mean ± SD	p-value	Mean ± SD	p-value
Number of children with ASD	1	144 (96.0)		3.787 ± 0.438	(0.015)	3.950 ± 0.491	(0.725)	4.257 ± 0.514	(0.348)	3.545 ± 0.483	(0.222)
	2	6 (4.0)		4.236 ± 0.406		4.021 ± 0.243		4.458 ± 0.510		3.792 ± 0.459	
Number of children without ASD	1	69 (46.0)		3.809 ± 0.348	(0.520)	3.957 ± 0.367	(0.528)	4.236 ± 0.460	(0.144)	3.467 ± 0.351	(0.119)
	2	70 (46.7)		3.824 ± 0.532		3.925 ± 0.582		4.332 ± 0.561		3.625 ± 0.595	
	3	11 (7.3)		3.659 ± 0.362		4.102 ± 0.443		4.023 ± 0.467		3.659 ± 0.302	
Age of children with ASD (Years)	≤ 2	18 (12.0)		3.787 ± 0.441	(0.001)	4.097 ± 0.547	(0.033)	4.403 ± 0.447	(0.005)	3.639 ± 0.260	(0.002)
	3 – 5	79 (52.7)		3.921 ± 0.338		4.000 ± 0.288		4.351 ± 0.432		3.563 ± 0.383	
	6 – 10	38 (25.3)		3.735 ± 0.231		3.905 ± 0.323		4.164 ± 0.436		3.664 ± 0.488	
	11 – 18	15 (10.0)		3.389 ± 0.780		3.650 ± 1.113		3.900 ± 0.855		3.133 ± 0.906	
Gender of children with ASD	Male	94 (62.7)		3.887 ± 0.363	(0.003)	3.957 ± 0.464	(0.872)	4.330 ± 0.418	(0.045)	3.350 ± 0.416	(0.246)
	Female	56 (37.3)		3.668 ± 0.530		3.944 ± 0.518		4.156 ± 0.418		3.496 ± 0.557	
Severity of ASD symptoms	Mild	42 (28.0)		3.808 ± 0.402	(0.006)	3.988 ± 0.372	(0.789)	4.179 ± 0.446	(0.001)	3.482 ± 0.328	(0.511)
	Moderate	96 (64.0)		3.852 ± 0.428		3.945 ± 0.510		4.357 ± 0.522		3.586 ± 0.533	
	Severe	12 (8.0)		3.424 ± 0.557		3.885 ± 0.623		3.833 ± 0.417		3.563 ± 0.285	

### Influence of impact of COVID-19 on family eating behavior

A multiple linear regression was used to test the impact of COVID-19 on the family’s eating behavior (**Tables 8**, **9**). This resulted in a significant model, F(1,148) = 10.670, p <.01, R2 = 0.067. The individual predictors were then scrutinized further and the outcomes indicated that while Impact of COVID-19 to family was a significant predictor (t=3.267, p<0.001), the overall facets of family ASD status were not significant predictors. The number of children with ASD was found to be a significant predictor in the early stages of the regression (t=2.048, p<0.05), but its impact was reduced when the other variables were added to the regression analysis.

**Table 8:** Model summary for influence of impact of COVID-19 on family eating behavior.

Model	R	R Square	Adjusted R Square	Std. Error of the Estimate	R Square Change	Change Statistics			
						F Change	df1	df2	Sig. F Change
1	0.259	0.067	0.061	0.969	0.067	10.670	1	148	0.001
2	0.305	0.093	0.081	0.959	0.026	4.195	1	147	0.042
3	0.306	0.093	0.075	0.962	0.000	0.052	1	146	0.821
4	0.307	0.094	0.069	0.965	0.001	0.127	1	145	0.723
5	0.327	0.107	0.076	0.961	0.013	2.093	1	144	0.150
6	0.328	0.108	0.070	0.964	0.000	0.057	1	143	0.811

**Table 9:** Path Coefficient for influence of impact of COVID-19 on family eating behavior.

Model	Independent variables	Unstandardized Coefficients		Standardized Coefficients	t	Sig.
		B	Std. Error	Beta		
1	(Constant)	0.000	0.079		0.000	1.000
	Impact of COVID-19 to family	0.259	0.079	0.259	3.267	0.001
2	(Constant)	0.000	0.078		0.000	1.000
	Impact of COVID-19 to family	0.241	0.079	0.241	3.046	0.003
	Number of children with ASD	0.162	0.079	0.162	2.048	0.042
3	(Constant)	0.000	0.079		0.000	1.000
	Impact of COVID-19 to family	0.242	0.080	0.242	3.045	0.003
	Number of children with ASD	0.161	0.079	0.161	2.028	0.044
	Number of children without ASD	-0.018	0.079	-0.018	-0.227	0.821
4	(Constant)	0.000	0.079		0.000	1.000
	Impact of COVID-19 to family	0.238	0.081	0.238	2.940	0.004
	Number of children with ASD	0.162	0.080	0.162	2.031	0.044
	Number of children without ASD	-0.011	0.082	-0.011	-0.138	0.890
	Age of children with ASD	-0.029	0.082	-0.029	-0.356	0.723
5	(Constant)	0.000	0.078		0.000	1.000
	Impact of COVID-19 to family	0.231	0.081	0.231	2.854	0.005
	Number of children with ASD	0.153	0.080	0.153	1.925	0.056
	Number of children without ASD	0.006	0.082	0.006	0.076	0.940
	Age of children with ASD	-0.029	0.082	-0.029	-0.359	0.720
	Gender of children with ASD	-0.116	0.080	-0.116	-1.447	0.150
6	(Constant)	0.000	0.079		0.000	1.000
	Impact of COVID-19 to family	0.230	0.081	0.230	2.833	0.005
	Number of children with ASD	0.153	0.080	0.153	1.914	0.058
	Number of children without ASD	0.002	0.084	0.002	0.024	0.981
	Age of children with ASD	-0.029	0.082	-0.029	-0.351	0.726
	Gender of children with ASD	-0.116	0.080	-0.116	-1.448	0.150
	Severity of ASD symptoms	0.019	0.081	0.019	0.239	0.811

## Influence of impact of COVID-9 on family daily routines

### Influence of impact of COVID-19 on family dinner time routines

A multiple linear regression was used to test the impact of COVID-19 on the family’s dinner time routines (**Tables 10**, **11**). This again resulted in a significant model, F(1, 148) = 4.460, p < 0.05, R2 = 0.036. The individual predictors were then scrutinized further and the outcomes indicated that while Impact of COVID-19 to family was a significant predictor (t=2.337, p<0.05), the facets of family ASD status were not significant predictors.

**Table 10:** Model summary for influence of impact of COVID-19 on family dinner time routines.

Model	R	R Square	Adjusted R Square	Std. Error of the Estimate	R Square Change	Change Statistics			
						F Change	df1	df2	Sig. F Change
1	0.189	0.036	0.029	0.9854	0.036	5.460	1	148	0.021
2	0.189	0.036	0.023	0.9886	0.000	0.020	1	147	0.888
3	0.195	0.038	0.018	0.9909	0.002	0.339	1	146	0.561
4	0.197	0.039	0.012	0.9939	0.001	0.114	1	145	0.736
5	0.197	0.039	0.005	0.9973	0.000	0.004	1	144	0.948
6	0.244	0.059	0.020	0.9899	0.021	3.154	1	143	0.078

**Table 11:** Path Coefficient for influence of impact of COVID-19 on family dinner time routines.

Model	Independent variables	Unstandardized Coefficients		Standardized Coefficients	t	Sig.
		B	Std. Error	Beta		
1	(Constant)	0.000	0.080		0.000	1.000
	Impact of COVID-19 to family	0.189	0.081	0.189	2.337	0.021
2	(Constant)	0.000	0.081		0.000	1.000
	Impact of COVID-19 to family	0.187	0.082	0.187	2.298	0.023
	Number of children with ASD	0.012	0.082	0.012	0.141	0.888
3	(Constant)	0.000	0.081		0.000	1.000
	Impact of COVID-19 to family	0.183	0.082	0.183	2.230	0.027
	Number of children with ASD	0.014	0.082	0.014	0.168	0.866
	Number of children without ASD	0.048	0.082	0.048	0.583	0.561
4	(Constant)	0.000	0.081		0.000	1.000
	Impact of COVID-19 to family	0.187	0.083	0.187	2.249	0.026
	Number of children with ASD	0.013	0.082	0.013	0.159	0.874
	Number of children without ASD	0.041	0.084	0.041	0.487	0.627
	Age of children with ASD	0.029	0.084	0.029	0.338	0.736
5	(Constant)	0.000	0.081		0.000	1.000
	Impact of COVID-19 to family	0.188	0.084	0.188	2.241	0.027
	Number of children with ASD	0.013	0.083	0.013	0.163	0.871
	Number of children without ASD	0.040	0.085	0.040	0.470	0.639
	Age of children with ASD	0.029	0.085	0.029	0.337	0.736
	Gender of children with ASD	0.005	0.083	0.005	0.066	0.948
6	(Constant)	0.000	0.081		0.000	1.000
	Impact of COVID-19 to family	0.194	0.083	0.194	2.330	0.021
	Number of children with ASD	0.016	0.082	0.016	0.196	0.845
	Number of children without ASD	0.072	0.087	0.072	0.832	0.407
	Age of children with ASD	0.025	0.084	0.025	0.291	0.771
	Gender of children with ASD	0.009	0.082	0.009	0.114	0.909
	Severity of ASD symptoms	-0.148	0.083	-0.148	-1.776	0.078

### Influence of impact of COVID-19 on family morning routines

A multiple linear regression was used to test the impact of COVID-19 on the family’s morning time routines (**Tables 12**, **13**). This again resulted in a significant model, F(1, 148) = 5.769, p < 0.05, R2 = 0.038. The individual predictors were then scrutinized further and the outcomes indicated that while Impact of COVID-19 to family was a significant predictor (t=2.402, p<0.05), the facets of family ASD status were not significant predictors.

**Table 12:** Model summary for the impact of COVID-19 on family morning routines.

Model	R	R Square	Adjusted R Square	Std. Error of the Estimate	R Square Change	Change Statistics			
						F Change	df1	df2	Sig. F Change
1	0.194	0.038	0.031	0.984	0.038	5.769	1	148	0.018
2	0.208	0.043	0.030	0.985	0.006	0.885	1	147	0.348
3	0.210	0.044	0.024	0.988	0.001	0.120	1	146	0.729
4	0.222	0.049	0.023	0.988	0.005	0.810	1	145	0.370
5	0.222	0.049	0.016	0.992	0.000	0.005	1	144	0.946
6	0.226	0.051	0.011	0.994	0.002	0.260	1	143	0.611

**Table 13:** Path Coefficient for the impact of COVID-19 on family morning routines.

Model	Independent variables	Unstandardized Coefficients		Standardized Coefficients	t	Sig.
		B	Std. Error	Beta		
1	(Constant)	0.000	0.080		0.000	1.000
	Impact of COVID-19 to family	0.194	0.081	0.194	2.402	0.018
2	(Constant)	0.000	0.080		0.000	1.000
	Impact of COVID-19 to family	0.185	0.081	0.185	2.278	0.024
	Number of children with ASD	0.076	0.081	0.076	0.941	0.348
3	(Constant)	0.000	0.081		0.000	1.000
	Impact of COVID-19 to family	0.182	0.082	0.182	2.230	0.027
	Number of children with ASD	0.078	0.082	0.078	0.953	0.342
	Number of children without ASD	0.028	0.081	0.028	0.347	0.729
4	(Constant)	0.000	0.081		0.000	1.000
	Impact of COVID-19 to family	0.170	0.083	0.170	2.056	0.042
	Number of children with ASD	0.080	0.082	0.080	0.977	0.330
	Number of children without ASD	0.046	0.084	0.046	0.546	0.586
	Age of children with ASD	-0.076	0.084	-0.076	-0.900	0.370
5	(Constant)	0.000	0.081		0.000	1.000
	Impact of COVID-19 to family	0.170	0.083	0.170	2.041	0.043
	Number of children with ASD	0.079	0.082	0.079	0.966	0.336
	Number of children without ASD	0.046	0.085	0.046	0.548	0.585
	Age of children with ASD	-0.076	0.084	-0.076	-0.897	0.371
	Gender of children with ASD	-0.006	0.083	-0.006	-0.068	0.946
6	(Constant)	0.000	0.081		0.000	1.000
	Impact of COVID-19 to family	0.172	0.084	0.172	2.055	0.042
	Number of children with ASD	0.080	0.082	0.080	0.972	0.332
	Number of children without ASD	0.056	0.087	0.056	0.641	0.523
	Age of children with ASD	-0.077	0.085	-0.077	-0.908	0.365
	Gender of children with ASD	-0.004	0.083	-0.004	-0.054	0.957
	Severity of ASD symptoms	-0.043	0.084	-0.043	-0.510	0.611

### Influence of impact of COVID-19 on family bedtime routines

A multiple linear regression was used to test the impact of COVID-19 on the family’s bed time routines (**Tables 14**, **15**). This again resulted in a significant model, F(1, 148) = 31.986, p < 0.01, R2 = 0.178. The individual predictors were then scrutinized further and the outcomes indicated that while Impact of COVID-19 to family was a significant predictor (t=5.656, p<0.001), the facets of family ASD status were not significant predictors apart from Number of children without ASD (t=2.819, p<0.05).

**Table 14:** Model summary for the impact of COVID-19 on family bedtime routines.

Model	R	R Square	Adjusted R Square	Std. Error of the Estimate	R Square Change	Change Statistics			
						F Change	df1	df2	Sig. F Change
1	0.422	0.178	0.172	0.910	0.178	31.986	1	148	0.000
2	0.426	0.182	0.171	0.911	0.004	0.734	1	147	0.393
3	0.488	0.238	0.222	0.882	0.056	10.762	1	146	0.001
4	0.488	0.238	0.217	0.885	0.000	0.001	1	145	0.982
5	0.489	0.240	0.213	0.887	0.002	0.305	1	144	0.581
6	0.493	0.243	0.211	0.888	0.003	0.609	1	143	0.436

**Table 15:** Path Coefficient for the impact of COVID-19 on family bedtime routines.

Model	Independent variables	Unstandardized Coefficients		Standardized Coefficients	t	Sig.
		B	Std. Error	Beta		
1	(Constant)	0.000	0.074		0.000	1.000
	Impact of COVID-19 to family	0.422	0.075	0.422	5.656	0.000
2	(Constant)	0.000	0.074		0.000	1.000
	Impact of COVID-19 to family	0.414	0.075	0.414	5.516	0.000
	Number of children with ASD	0.064	0.075	0.064	0.857	0.393
3	(Constant)	0.000	0.072		0.000	1.000
	Impact of COVID-19 to family	0.393	0.073	0.393	5.375	0.000
	Number of children with ASD	0.076	0.073	0.076	1.040	0.300
	Number of children without ASD	0.238	0.073	0.238	3.281	0.001
4	(Constant)	0.000	0.072		0.000	1.000
	Impact of COVID-19 to family	0.393	0.074	0.393	5.292	0.000
	Number of children with ASD	0.076	0.073	0.076	1.035	0.302
	Number of children without ASD	0.238	0.075	0.238	3.175	0.002
	Age of children with ASD	0.002	0.075	0.002	0.023	0.982
5	(Constant)	0.000	0.072		0.000	1.000
	Impact of COVID-19 to family	0.395	0.075	0.395	5.303	0.000
	Number of children with ASD	0.079	0.073	0.079	1.071	0.286
	Number of children without ASD	0.232	0.076	0.232	3.052	0.003
	Age of children with ASD	0.002	0.075	0.002	0.023	0.981
	Gender of children with ASD	0.041	0.074	0.041	0.553	0.581
6	(Constant)	0.000	0.073		0.000	1.000
	Impact of COVID-19 to family	0.393	0.075	0.393	5.259	0.000
	Number of children with ASD	0.078	0.074	0.078	1.056	0.293
	Number of children without ASD	0.219	0.078	0.219	2.819	0.005
	Age of children with ASD	0.003	0.076	0.003	0.045	0.965
	Gender of children with ASD	0.039	0.074	0.039	0.531	0.597
	Severity of ASD symptoms	0.058	0.075	0.058	0.780	0.436

### Parents’ comments

About (35%) of parents’ comments at the end of the questionnaire indicated that their children lost weight compared to the period before the pandemic and they had encountered different challenges due to the COVID-19 situation, specifically with regard to procurement of the specific brands of food preferred by their children with ASD. Moreover, 40% indicated that dealing with multiple children with ASD requires planning and advice should be less cumbersome.

## Discussion

To the best of the researcher’s knowledge, this is one of the first studies placing emphasis on the eating behavior and daily routines of children with ASD in Saudi Arabia during the coronavirus outbreak. Overall, the study found that parents perceived that the behaviors of their children with ASD concerning these had deteriorated due to the pandemic-related change in their situations.

### Current status of eating behavior and daily routines

The study found that the children’s eating behavior had significantly worsened in comparison to their normal state in the pre-pandemic context. Eating challenges are a characteristic of most children with ASD (26, 27). The aspects of eating behavior that had changed the most were rejection of food based on color or shape, and texture and increased consumption of only certain kinds of food. This was in line with prior studies which have highlighted that selectivity of food by type and texture are common in children with ASD (32, 40). Moreover, the findings confirm that the food repertoire of a child with ASD does not typically change with time (14). This is further confirmed by the finding that the facets of eating behavior that had changed the least were related to pica; smelling foods prior to eating; and not mixing foods prior to eating.

Further, the study found that the daily routines of the children with ASD had also worsened. These findings were in line with Altable (11) and Eshraghi et al. (21), who highlighted that the daily routines of children with ASD have been impacted due to the pandemic in different ways. In particular, the parents perceived that the children’s morning routines were the most impacted, followed by the dinner-time routines and bedtime routines. It is possible that the dinner-time and bedtime routines were not impacted as much as the morning routines because the morning routines would usually have involved the child getting ready (or being readied) to leave the house to attend school or an intervention session. Prior research (22, 62, 63) has highlighted that bedtime routines were typically the most structured whereas dinnertime routines were more disordered, though children with ASD can find it difficult to participate in such routines. Nevertheless, it could be inferred that the disruption of the routines of the child with ASD had consequences for the families of the participants of the study.

### Relationships between impact of COVID-19 to family, eating behavior, daily routines, and family ASD status

The study found that the change in the eating behavior of a child with ASD was found to differ significantly based on the number of children with ASD, age of children with ASD, gender of children with ASD, and severity of their ASD symptoms. Consistent with prior studies, the changes to the eating behavior of the children with ASD was related to the severity of their ASD symptoms (17) and also their age (14, 16, 47). Eating behavior was also related to the gender of the children (57, 58). Changes to the eating behavior of the children with ASD was also related to the number of children with ASD in the family. To the researcher’s best knowledge, this aspect has not been previously explored in research.

Moreover, on the lines of the findings of Huxham et al. (19) and Padmanabhan and Shroff (18), this study found that the facets of eating behavior that the children appeared to struggle with included their preference for foods of certain colors, shapes, textures, kinds, or brands, and for food to be presented in a particular manner. Other eating behavior had also deteriorated, such as pocketing of food in contrast to swallowing it (20).

The study found that the worsening of dinner-time routines was related to the age of the child with ASD but not to their gender or the severity of their symptoms. Also, it was not related to the number of children with and without ASD in the family. The worsening of morning routines was found to be related to the age of the child with ASD, their gender, and the severity of symptoms, but not to number of children with and without ASD in the family. Finally, the parents’ perceptions of the bedtime routines were found to be related to the age of the child with ASD. The study’s findings on the relationship between the age of the children with ASD and their routines were on the lines of Marquenie et al. (22) and Stoppelbein et al. (66). Moreover, the scrutiny on the associations between gender, ASD severity, and routines does not seem to have received much consideration although Stoppelbein et al. (66) did not find a significant relationship between gender and child routines and severity and child routines.

Furthermore, from the parents’ notes, this study found that the COVID situation had made it difficult for parents to obtain the brands of food their children preferred. This could have contributed to the worsening of the children’s eating behavior and consequently some of the activities involved in their mealtime routines.

## Conclusion

This study found that the eating behavior and daily routines of children with ASD in Saudi Arabia considerably worsened during the coronavirus pandemic. Given that the ongoing and the attempts of governments in Saudi Arabia and across the world to contain the situation have met with mixed success, it is possible that this situation may be returned. In such case, the closing of educational institutions for children with ASD. In this context, this study highlights the impacts on the behavior of children with ASD due to changes in their regular routines, which might include regular attendance at mainstream or special schools and a variety of interventions.

Moreover, by obtaining the perceptions of the parents of children with ASD across different age groups (≤18 years) and of mixed gender and varying levels of severity of ASD symptoms, this study highlights the fact that changes in schedule or context do impact children with ASD regardless of their age, gender, or ASD severity. A downstream impact of the effects on children with ASD is the impact on their parents’ wellbeing (25). Consequently, it is imperative that measures be taken to help children with ASD deal with such epidemic-related situations and to prepare them for the changes that may lie ahead.

### Limitations of the study

This study is not without limitations. Firstly, the study was undertaken after pandemic-related precautions were implemented in Saudi Arabia. Consequently, it is possible that some measures may already have been undertaken to mitigate the impact on children with ASD. Future researchers could seek to overcome these limitations by undertaking a study with a broader sample and by performing a longitudinal study where the impacts on children with ASD are scrutinized at different points in the pandemic timeline. Additionally, it appeared that the parents were experiencing some form of fatigue in responding to the questionnaire, perhaps due to the surfeit of similar studies being undertaken across the globe.

Moreover, no data were explicitly collected related to the pre-pandemic status of the children of the participating families. A future researcher could rectify this situation. An additional facet could be the involvement of external stakeholders, such as dietitians, teachers and therapists, to provide further insights regarding the eating behavior and routines of the children with ASD.

### Implications for research and practice

The findings of the study indicate that it is necessary for support for children with ASD to be expanded and reconsidered during the spread of any pandemic. The following recommendations are made in this regard:

i) Parents, teachers, behavior analysts, psychologists, dieticians, or nutritionists and others associated with the welfare of persons with ASD in Saudi Arabia must take a range of measures to help them deal with the pandemic-related changes to their schedule. For instance, parents could be trained to provide behavioral support and reinforcement (24).
ii) Behavior analysts with dieticians and/or nutritionists could collaborate with parents to adapt or design interventions that can be implemented at home by either or both parents.
iii) The Saudi Ministry of Health and Ministry of Education could initiate programs to oversee the training of parents and other caretakers of children with ASD during the pandemic and/or lockdown and promote the use of online interventions to support children of different age groups.
iv) The Ministry of Health and Ministry of Education could also facilitate the development of more virtual helplines to ensure that all parents of children with ASD can access varied sources of assistance such as behavior analysts, dieticians, and nutritionists.

## Data availability statement

The original contributions presented in the study are included in the article/supplementary material. Further inquiries can be directed to the corresponding author.

## Ethics statement

This study was carried out in accordance with the Declaration of Helsinki and with the approval of the the Applied Medical Science committee at University of Taibah no, 2020/52/201/CLN. The studies were conducted in accordance with the local legislation and institutional requirements. The participants provided their written informed consent to participate in this study.

## Author contributions

MA: Conceptualization, Data curation, Formal analysis, Investigation, Methodology, Supervision, Validation, Visualization, Writing – original draft, Writing – review & editing.
